# A comprehensive measure of Golgi sphingolipid flux using NBD C_6_-ceramide: evaluation of sphingolipid inhibitors

**DOI:** 10.1016/j.jlr.2024.100584

**Published:** 2024-06-24

**Authors:** Allen H. Lee, Justin M. Snider, Sitapriya Moorthi, Nicolas Coant, Magali Trayssac, Daniel Canals, Christopher J. Clarke, Chiara Luberto, Yusuf A. Hannun

**Affiliations:** 1 Department of Medicine, Stony Brook University, Stony Brook, NY, USA; 2 Department of Physiology and Biophysics, Stony Brook University, Stony Brook, NY, USA; 4 Department of Medicine, The Northport Veterans Affairs Hospital, Northport, NY, USA; 3 Department of Pathology, Stony Brook University, Stony Brook, NY, USA

**Keywords:** NBD-ceramide, Golgi apparatus, sphingolipid metabolism, CERK, PDMP, fenretinide, HPLC

## Abstract

Measurements of sphingolipid metabolism are most accurately performed by LC-MS. However, this technique is expensive, not widely accessible, and without the use of specific probes, it does not provide insight into metabolic flux through the pathway. Employing the fluorescent ceramide analogue NBD-C_6_-ceramide as a tracer in intact cells, we developed a comprehensive HPLC-based method that simultaneously measures the main nodes of ceramide metabolism in the Golgi. Hence, by quantifying the conversion of NBD-C_6_-ceramide to NBD-C_6_-sphingomyelin, NBD-C_6_-hexosylceramides, and NBD-C_6_-ceramide-1-phosphate (NBD-C1P), the activities of Golgi resident enzymes sphingomyelin synthase 1, glucosylceramide synthase, and ceramide kinase (CERK) could be measured simultaneously. Importantly, the detection of NBD-C1P allowed us to quantify CERK activity in cells, a usually difficult task. By applying this method, we evaluated the specificity of commonly used sphingolipid inhibitors and discovered that 1-phenyl-2-decanoylamino-3-morpholino-1-propanol, which targets glucosylceramide synthase, and fenretinide (4HPR), an inhibitor for dihydroceramide desaturase, also suppress CERK activity. This study demonstrates the benefit of an expanded analysis of ceramide metabolism in the Golgi, and it provides a qualitative and easy-to-implement method.

Over the past 3-4 decades, many key sphingolipids (SLs) have been implicated in diseases and have been found to regulate a number of biological responses including apoptosis, migration, proliferation, senescence, and angiogenesis, among others (1). These discoveries have propelled interest in SL metabolism which is now appreciated for its biologic functions but also for its complexity and metabolic interconnectedness. Much of our current understanding of SL metabolism has been revealed through the use of various analytical methods and molecular probes. Classically, the cellular activity of SL metabolizing enzymes has been measured by labeling with precursors followed by analysis using TLC or HPLC alone or LC-MS/MS. While TLC suffers from low sensitivity, it typically offers excellent separation of many lipid classes utilizing solvent systems not traditionally used in LC applications. On the other hand, LC-MS/MS is more sensitive and specific in the detection of molecular species but is much less accessible and more expensive. In contrast, HPLC provides both sufficient sensitivity and ease of use.

N-[6-[(7-Nitro-2-1,3-benzoxadiazol-4-yl) amino] dodecanoyl] (NBD)-C_6_-ceramide (Cer) has been used as a precursor to measure the activity of SL enzymes acting on Cer, but it has also been extensively used to study Golgi sphingolipid trafficking and metabolism. Work by Lipsky and Pagano showed that NBD-C_6_-Cer is quickly taken up and concentrates in the Golgi, where it is converted into other metabolites in a manner that bypasses the endoplasmic reticulum (ER), before eventually being delivered to the plasma membrane (2). The same authors also showed that NBD-C_6_-Cer did not affect cell viability and that the bulky nitrobenzyl group did not alter substrate recognition since NBD-C_6_-Cer and radiolabeled Cer were similarly metabolized (3). Of the main branches of Cer metabolism, the key sphingolipid enzymes whose activity could be measured by monitoring their NBD-sphingolipid products are Golgi resident enzymes, most notably sphingomyelin synthase 1 (SMS1), glucosylceramide synthase (GCS), and ceramide kinase (CERK), which generate sphingomyelin, glucosylceramide, and ceramide 1-phosphate (C1P), respectively (4, 5, 6, 7). NBD-C_6_-Cer has been used in situ (where the compound is added to intact cells) for determining the IC50 of different GCS inhibitors (8, 9, 10, 11). In 2010, Gupta *et al.* developed a TLC method for quantifying GCS activity in situ (12). Recently NBD-C_6_-Cer was used in situ for measuring GCS activity and helped to elucidate the role of ABCC10 in the synthesis and efflux of glucosyl-ceramide in Huh-7 cells (13).

CERK is the main enzyme that phosphorylates Cer and has been linked with inflammatory processes including prostaglandin synthesis (14, 15, 16). Both CERK and its product C1P have also been shown to promote cell proliferation and migration (17, 18, 19, 20). However, CERK activity has been particularly difficult to study and measure with in vitro CERK activity currently being measured using a plate reader-based fluorescent assay, TLC, or LC/MS/MS (4, 7, 21). Similarly, due to the low amount of C1P produced from Cer compared to other complex sphingolipids, it can also be challenging to detect the minute changes in C1P by fluorescence without mass spectrometry analysis. As such, studying CERK in cell-based systems has been challenging, and more refined methods are needed.

In this work, we develop a facile assay to measure the flux of NBD-C_6_-Cer into its Golgi products and to probe the activity of key enzymes of sphingolipid metabolism simultaneously, focusing on SMSs, GCS, and CERK. This newly developed HPLC method provides a cell-based assay for monitoring CERK activity. It also afforded an examination of the specificity of several common inhibitors of sphingolipid enzymes. This led us to discover that 1-phenyl-2-decanoylamino-3-morpholino-1-propanol (PDMP) and fenretinide (4HPR)—previously reported to target GCS and dihydroceramide desaturase 1 (DES1), respectively—also showed significant inhibitory effects on CERK activity.

## Materials and methods

### Chemicals and reagents

MCF7 cells were from American Type Culture Collection (ATCC, Manassas, VA). Fetal bovine serum, RPMI, and clonase II were from life technologies (Carlsbad, CA). XtremeGene was from Roche (Basil, CH). Polybrene was from Sigma-Aldrich (St. Louis, MO). Blasticidin was from InvivoGen (San Diego, CA). NBD-C_6_-Cer, NBD-C_6_-glucosylceramide, NBD-C_6_-sphingomyelin, and NBD-C_6_-C1P were purchased from Cayman Chemical (Ann Arbor, Michigan). Fatty acid free bovine serum albumin (BSA) was purchased from Gemini Bio (West Sacramento, CA). MS grade methanol, MS grade isopropanol, MS, grade ethyl acetate, MS grade water, chloroform, ammonium formate, and formic acid for lipid extraction and HPLC were from Thermo Fisher Scientific (Waltham, MA). The C8 column (3 μM particle, 4.6 × 150 mm) for HPLC was from Peeke Scientific (Redwood City, CA). NVP-231, HPA-12, PDMP, eliglustat, D-MAPP, and desipramine were purchased from Cayman Chemical (Ann Arbor Michigan). Enzastaurin was purchased from LC Labs (Woburn, MA), LMK-235 was purchased from Selleckchem (Houston, TX). Vorinostat and myriocin were purchased from Sigma-Aldrich (St. Louis, MO). C_6_-urea ceramide was purchased from Avanti Polar Lipids (Alabaster, AL). Fumonisin B1 was purchased from Enzo Life Sciences (Farmingdale, NY). 4HPR was purchased from MedKoo Biosciences (Durham, NC). PF-543 was purchased from Calbiochem (San Diego, CA). DMSO, lipofectamine RNAiMax, Opti-Mem, and all siRNA from Thermo Fisher Scientific. Hepes, KCl, MgCl_2_, glycerol, and DTT were purchased from Sigma-Aldrich (St. Louis, MO). ATP was purchased from Life Technologies (Carlsbad, CA).

### Cell lines and cell culture

MCF7 human breast adenocarcinoma cells were obtained from ATCC and cultured in RPMI media containing 10% fetal bovine serum. Cells were maintained in a humidified incubator at 37°C with 5% CO2 and were typically passaged every 3–4 days. Cells were regularly tested for mycoplasma contamination (Lonza).

### Generation of stable LacZ/CERK-overexpressing MCF-7 cells using lentivirus

To prepare lentiviral particles, it was first necessary to generate a viral CERK construct. For this, the pcDNA3.1 HisTOPO-hCERK plasmid (a gift from Charles Chalfant) was used as a template (22). The CERK cDNA was first cloned into pENTR-V5 (a gift from Eric Campeau; Addgene #17425) and then introduced into pLenti CMV/TO Blast DEST (a gift from Eric Campeau; Addgene #17451) by a recombinase reaction with LR Clonase II (Life Technologies) according to the manufacturer’s instructions. To generate viral particles, 2 × 10^6^ HEK293T cells were plated in 100 mm dishes and at 24 h were cotransfected with 1.5 μg of vesicular stomatitis virus G glycoprotein plasmid, 1.5 μg DVPR plasmid, and 1.5 μg of the CERK/LacZ pLenti plasmids using 13.5 ml Xtremegene (Roche) in 300uL OptiMem (Gibco). After 24 h, the media were replaced, and after a subsequent 48 h virus-containing media were collected, filtered, divided into 1 ml aliquots, and stored at −80°C. For infection, 5 × 10^5^ MCF-7 cells in 6 well dishes were plated into media containing the viral constructs and 10 μg/ml polybrene (Santa Cruz). Media were replaced after 48 h with selective media containing 20 μg/ml blasticidin (InvivoGen) and maintained under selective pressure for a week. For generation of a matching control cell line, pLenti-LacZ-blast was used.

### Inhibitor treatments on cells

Medium was replaced 1 h prior to treatment with inhibitors, and inhibitors were added directly to the media for 4 h before labeling with NBD-C_6_-Cer. The inhibitors NVP-231 (CERK), HPA-12 (ceramide transfer protein, CERT), PDMP (GCS), and eliglustat (GCS) were added to the media at final concentrations of 500 nM, 20 μM, 50 μM, and 50 nM, respectively, and DMSO or water was used as control. Inhibitors were ordered from Cayman Chemical.

### Transfection with siRNA

siRNAs were obtained from Thermo Fisher Scientific. When available, validated siRNAs were selected against the following targets: *CERK* (validated s34929), *UGCG* (validated s14643), *COL4AB3P* (CERT) (s19625), *SGMS1* (s48914), and *SGMS2* (s46644). All-Star negative control siRNA was obtained from Qiagen. The 2.5 × 10^5^ cells in 60 mm dishes with complete media were transfected with 20 nM siRNA at the time of plating using Lipofectamine RNAiMAX (Thermo Fisher Scientific) transfection reagent according to manufacturer’s protocol. Briefly, 3 ml of the siRNA in 150 ml of Opti-MEM was mixed with 3 μl of Lipofectamine RNAiMAX in 150 μl of Opti-MEM. Complexes were incubated at room temperature for 5–10 min at room temperature before being added dropwise to media in dishes, prior to the addition of cells. After 48 h, the media were replaced for 1 h prior to labeling with NBD-C_6_-Cer.

### NBD-C_6_-ceramide labeling and lipid extraction

NBD-C_6_-Ceramide (Cayman Chemical 62527) was conjugated to fatty acid-free BSA in PBS. Briefly 100 μL of 1 mM NBD-C_6_-Cer was dried down under nitrogen gas and redissolved in 200 μL of absolute ethanol. This was added to 1 ml of 0.34 mg/ml fatty acid-free BSA in PBS and vortexed. The resulting 100 μM NBD-C_6_-Cer/BSA complex was stored at −20°C. Roughly, 2.5 × 10^5^ cells in 60 mm dishes were labeled with NBD-C_6_-ceramide by direct addition of room temperature NBD-C_6_-Cer/BSA complex to cell culture medium (complete serum or reduced serum) at the indicated final concentrations. The cells were incubated for the duration of the indicated times with NBD-C_6_-ceramide in a humidified incubator at 37°C with 5% CO_2_ and kept in the dark. At the end of the incubation, media (2 ml) were collected, and lipids were extracted from media in 4 ml of 15:85 MS grade isopropanol: MS grade ethyl acetate. Cell lipids were collected as adapted from Snider *et al.* (23). The plates of cells were washed with room temperature PBS, scraped in 2 ml of 2:3 70% MS grade isopropanol in MilliQ water: MS grade ethyl acetate, and collected in a 15 ml conical tube. Another 2 ml of 2:3 70% isopropanol: ethyl acetate were added to plates and collected in the same 15 ml conical tube to collect residue lipids on the plate. One milliliter of cell lipid extract was dried under nitrogen gas and saved at −80°C for inorganic phosphate measurements. Media and cell lipid extracts were dried down under nitrogen gas and resuspended in 100 μl of MS grade methanol for subsequent HPLC analysis. MS grade solvents were purchased from Thermo Fisher Scientific.

### Lipid analysis by HPLC and fluorescence detection

An injection of 3 μl of extracted lipids was separated using an Agilent 1260 Quaternary HPLC System equipped with a Peeke Scientific C-8 column (3 μM particle, 4.6 × 150 mm). Column temperature of 30°C maximized intensity and integrity of analytes while maintaining baseline separation. All MS grade solvents for the following mobile phases were purchased from Thermo Fisher Scientific. Mobile phase A (MPA) consisted of MS grade water containing 0.2% (v/v) formic acid and 1 mM ammonium formate (pH 5.6). Mobile phase B (MPB) consisted of MS grade methanol containing 0.2% (v/v) formic acid and 1 mM ammonium formate (pH 5.6), and mobile phase C (MPC) consisted of MS grade acetonitrile containing 0.1% (v/v) formic acid. The chromatographic conditions were as follows: upon sample injection, the gradient was held constant for 3 min at 40% MPA, 40% MPB, and 20% MPC, then changed to 30% MPA, 65% MPB, and 5% MPC by 5 min, then changed to 10% MPA, 85% MPB, 5% MPC by 20 min, then to 1% MPA, 94% MPB, and 5% MPC by 24 min. The gradient was then brought back to 40% MPA, 40% MPB, and 20% MPC by 26 min and re-equilibrated for a total run time of 30 min. Fluorescence was measured with an Agilent G1321B detector with excitation and emissions set to 470 nm and 530 nm, respectively. The area under the corresponding NBD-sphingolipid peak was measured and normalized to total inorganic phosphate of the respective sample. Dried aliquots were set aside for inorganic phosphate measurements, reextracted (24), and assayed as described (25). Data were analyzed utilizing Openlab Agilent software.

### Low-throughput screening of inhibitors’ effects

MCF7-LacZ and MCF7-CERK cells (2.5 × 10^5^ cells, 60 mm dishes) were grown as described above for 48 h. Medium was replaced for 1 h and treatments were added directly to the media for 4 h prior to NBD-C_6_-Cer labeling, with the exception of desipramine which was added for 1 h prior to labeling. The final concentrations of the treatments are as follows: 1 μM enzastaurin (LC Labs), 2.5 μM LMK-235 (Selleckchem), 5 μM vorinostat (Sigma-Aldrich), 5 μM C_6_-urea ceramide (Avanti), 10 μM D-MAPP (Cayman Chemical), 10 μM desipramine (Cayman Chemical), 100 nM myriocin (Sigma-Aldrich), 50 μM fumonisin B1 (Enzo Life Sciences), 5 μM 4HPR (MedKoo Biosciences), and 100 nM PF-543 (Calbiochem). These concentrations were based on prior validation studies on effective concentrations against their intended targets in cells (26, 27, 28, 29, 30, 31, 32, 33, 34).

### Analysis of CERK activity by in vitro assay

As a protein source, the full-length human CERK was expressed in Sf9 cells, grown in Sf-900™ III SFM media (Invitrogen) at 28°C with agitation. Recombinant CERK protein was purified from the cellular extract through HisTrap excel column (GE HealthCare) using an AKTA FPLC system. CERK containing fractions were pooled, concentrated, and flash frozen. This purification method yields approximately 0.1 mg (∼0.01 enzymatic units; μmol/min) of purified protein from a 1-l SF9 culture (35). For substrate, the in vitro CERK assay used NBD-C_6_-Cer prepared in chloroform as a 1 mM stock in a glass tube. To prepare the assay solution, one nmol of C_6_-NBD substrate stock per reaction was dried down under nitrogen and resuspended in 50 μl of assay buffer (40 mM Hepes pH 7.4, 20 mM KCl, 30 mM MgCl2, 20% glycerol, 2 mM DTT, 2 mM ATP, 0.4 mg/ml fatty acid free BSA) and sonicated for 15 min. Fifty nanogram purified CERK was diluted to a volume of 50 μl, mixed with 50 μl of C_6_-NBD substrate solution, and incubated at 37 °C for 1 h. The reaction was terminated by addition of 200 μL chloroform:methanol (1:1) and centrifuged at 2000 g for 5 min. The organic phase was collected and dried, and the lipids were dissolved in 50 μl of methanol. From here, 10 μl of lipid extract was loaded on a Spectra 3 μm C8SR column (0.3 mm × 150 mm) and eluted in methanol/water. Fluorescent NBD-C_6_-C1P product was quantitated by peak area and compared to NBD-C_6_-C1P standards.

### Statistical analysis

Student’s *t* test was used to determine significant differences in the means of sample groups for comparison between two conditions. Significant differences for comparisons of three or more conditions were determined using a one-way ANOVA with Bonferroni post hoc test between all sample groups. Significance in comparisons involving two or more variables was determined by two-way ANOVA with Bonferroni post hoc tests. Graphpad Prism 7 (graphpad.com) was used to conduct all statistical tests. *P* < 0.05 was considered statistically significant for all experiments.

## Results

### Incorporation of NBD-C_6_-Cer into complex NBD-sphingolipids

**Fig. 1:**
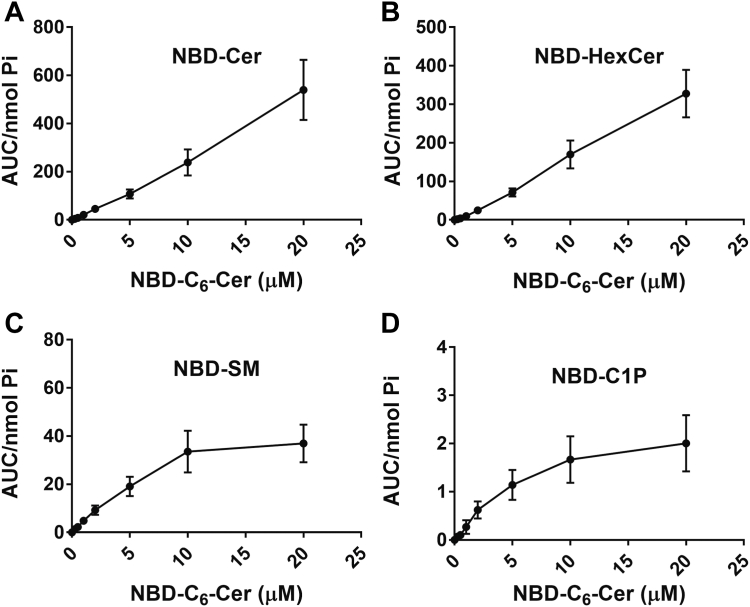
Dose-response of MCF7 treatment with NBD-C_6_-Cer. MCF7 cells were treated with varying doses of NBD-C_6_-Cer as shown for 1 h. Lipids were extracted and analyzed for (A) NBD-C_6_-Cer and its conversion into (B) NBD-HexCer; (C) NBD-SM; and (D) NBD-C1P by HPLC and fluorescence detection. Levels were normalized to total lipid phosphate. Results are expressed as mean ± SEM of the area under the curve (AUC) of detected peaks and are representative of at least three independent experiments. C1P, ceramide-1-phosphate; NBD, N-[6-[(7-Nitro-2-1,3-benzoxadiazol-4-yl)amino]dodecanoyl].

The known accumulation of NBD-C_6_-Cer in the Golgi suggested it as an ideal substrate for monitoring Golgi Cer flux. However, it was first necessary to assess the incorporation of NBD-C_6_-Cer into MCF7 cells and assess the enzymatic conversion of NBD-C_6_-Cer into NBD-C_6_-HexCer, NBD-C_6_-SM, and NBD-C_6_-C1P, respectively. For this, a dose-response of NBD-C_6_-Cer treatment (from 0 μM to 20 μM NBD-C_6_-Cer) was performed for 1 h, and the levels of NBD-C_6_-sphingolipids were measured. To identify the appropriate peaks for NBD-C_6_-Cer, NBD-C_6_-HexCer, NBD-C_6_-SM, and NBD-C_6_-C1P, individual NBD standards for each product were combined and separated by the same methodology (supplemental Fig. S1). As can be seen, NBD-C_6_-Cer was incorporated into the cells in a linear fashion (Fig. 1A), and generation of NBD-C_6_-HexCer was also linear over this dose range (Fig. 1B). In contrast, while conversion of NBD-C_6_-Cer into NBD-C_6_-SM and NBD-C_6_-C1P initially increased as the dose of NBD-C_6_-Cer increased, the rate of conversion for each started decreasing at 10 μM NBD-C_6_-Cer (SM) and 5 μM NBD-C_6_-Cer (C1P), respectively (Fig. 1C, D), suggesting saturation of flux through SMS and CERK at those concentrations. Taking these results together, to assess enzymatic activities in situ, treatment of MCF-7 cells with NBD-C_6_-Cer for 1 h should be at concentrations lower than 5 μM as any changes in sphingolipid metabolism at this concentration would not be confounded by saturation of the enzymes or by significant biologic activities of NBD-C_6_-Cer. Therefore, all following experiments were performed with 1 μM NBD-C_6_-Cer treatment.

**Fig. 2:**
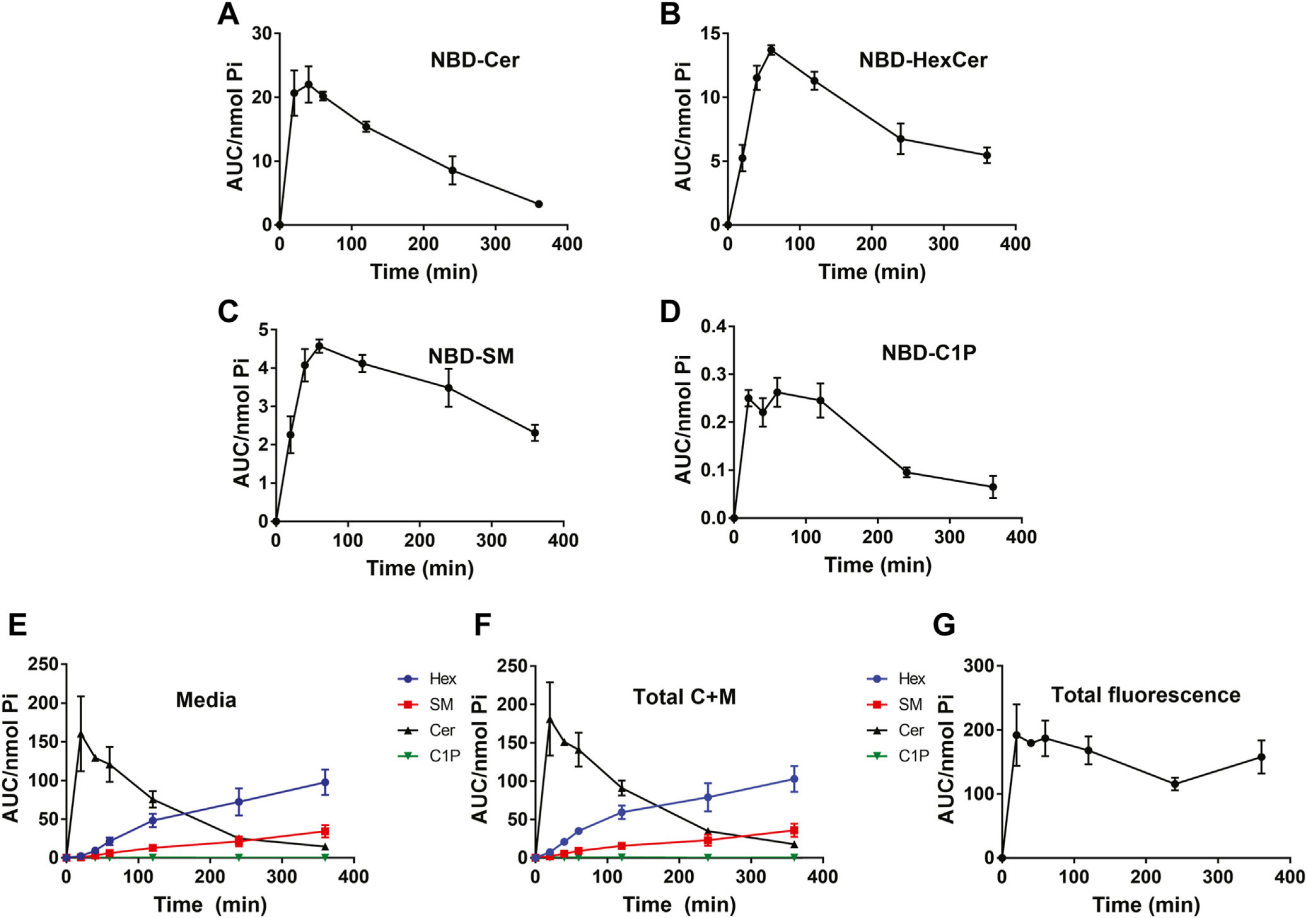
Time course of MCF7 treatment with NBD-Cer. MCF7 cells were treated with 1 μM NBD-C_6_-Cer over the course of 6 h. Lipids were extracted and analyzed for (A) NBD-C_6_-Cer and its conversion to (B) NBD-HexCer, (C) NBD-SM, and (D) NBD-C1P by HPLC and fluorescence detection. Lipids in the media of these cells were extracted and analyzed for (E) NBD-Cer, NBD-HexCer, NBD-SM, and NBD-C1P by HPLC and fluorescence detection. (F) Measurements of the total of NBD-Cer, NBD-HexCer, NBD-SM, and NBD-C1P from cells and media. (G) Total NBD-Fluorescence in all the species, combining cell and media, was measured across the time course. Levels of NBD-sphingolipids were normalized to total lipid phosphate. Results are expressed as mean ± SEM of the AUC of detected peaks and are representative of at least three independent experiments. Student’s *t* test was used for statistics. AUC, area under the curve; C1P, ceramide-1-phosphate; NBD, N-[6-[(7-Nitro-2-1,3-benzoxadiazol-4-yl)amino]dodecanoyl].

Having established an optimal dose of NBD-C_6_-Cer, it was important to determine an optimal time of incubation for maximal detection of the three products. For this, a time course at 1 μM NBD-C_6_-Cer treatment was performed in order to establish the rates of incorporation of NBD-C_6_-Cer and its conversion to NBD-C_6_-HexCer, NBD-C_6_-SM, and NBD-C_6_-C1P (Fig. 2A–D). Measuring the NBD-sphingolipids from 0 to 360 min, results showed that most complex sphingolipids saw peak fluorescence intensity in the cells at 60 min, the time point after which the products started to appear in the media (Fig. 2E). Total levels of individual complex NBD-sphingolipids (cells plus media) accumulated in a linear fashion, suggesting that the activities of the sphingolipid enzymes are linear within this time frame (Fig. 2F). As expected, total fluorescence was maintained throughout the time course (Fig. 2G), suggesting lack of substantial diversion of the NBD label into other metabolites. From these results, for all subsequent experiments, 60 min was chosen as the optimal time of incubation as the cellular levels of the NBD products (NBD-C_6_-HexCer, NBD-C_6_-SM, and NBD-C_6_-C1P) reflect the activities of the respective enzymes (with minimal release of the products in the media) for maximal accumulation of NBD-C_6_-Cer and production of the NBD products.

### Disruption of Golgi sphingolipid flux by pharmacological and genetic approaches

**Fig. 3:**
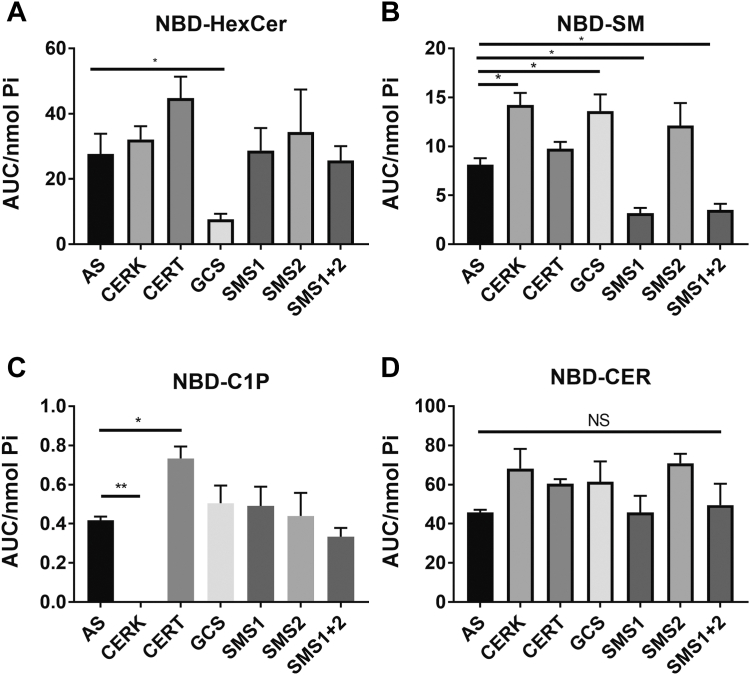
Measurement of NBD-sphingolipids in siRNA-treated MCF7 cells. MCF7 cells were transfected for 48 h with all stars (AS) scrambled siRNA or siRNA for CERK, CERT, GCS, SMS1, SMS2, or SMS1+2. Following transfection, the cells were treated with 1 μM NBD-Cer for 1 h. Lipids were extracted and analyzed for (A) NBD-HexCer, (B) NBD-SM, (C) NBD-C1P, and (D) NBD-Cer by HPLC and fluorescence detection. Levels were normalized to total lipid phosphate. Results are expressed as mean ± SEM of the AUC of detected peaks and are representative of at least three independent experiments. One-way ANOVA was used for statistics (∗ = *P* < 0.05, ∗∗ = *P* < 0.005). AUC, area under the curve; CERT-ceramide transfer protein; CERK, ceramide kinase; GCS, glucosylceramide synthase; NBD, N-[6-[(7-Nitro-2-1,3-benzoxadiazol-4-yl)amino]dodecanoyl]; SMS1, sphingomyelin synthase 1.

Having established assay conditions as discussed above, it was important to validate that the assay was sensitive enough for detecting alterations in enzymatic activity. For this, the cells were subjected to siRNA knockdown of the key enzymes in the conversion of NBD-C_6_-Cer. Transfection of siRNA was conducted at the time of plating 48 h prior to 1 h NBD-C_6_-Cer treatment. For validation of knockdown, RNA was extracted from parallel siRNA-treated plates. As can be seen (supplemental Fig. S2), mRNA of the target genes of interest were efficiently knocked down (at least 85% knockdown) by their respective siRNA. Of note, some siRNA had effects on the expression of other genes with knockdown of SMS1 leading to increased CERT and SMS2 mRNA (supplemental Fig. S2A, E), the knockdown of SMS2 causing a decrease in GCS mRNA and increase in SMS1 mRNA (supplemental Fig. S2C, D), and the knockdown of CERK also decreasing GCS mRNA (supplemental Fig. S2C). Although some of these effects could be anticipated and are consistent with prior studies e.g. SMS1 and SMS2 knockdown promoting expression of the other isoform, the reasons for other such changes are not clear. Importantly, knockdown of each target sphingolipid enzyme led to a profound decrease in their respective products (Fig. 3A–C) although in all cases this did not result in alterations in NBD-C_6_-Cer levels (Fig. 3D). Importantly, only the knockdown of GCS resulted in a 3-fold decrease of NBD-C_6_-HexCer (Fig. 3A) while knockdown of SMS1 but not SMS2 led to a 2-fold reduction in NBD-C_6_-SM (Fig. 3B)—consistent with the literature that SMS1 is localized to the Golgi while SMS2 is primarily localized to plasma membrane (36). These results further enforce that these relatively acute kinetics are a reflection of primarily Golgi metabolism of NBD-C_6_-Cer. Interestingly, as with mRNA expression, there were some broader impacts of specific siRNAs on NBD products with the knockdown of either CERK or GCS also resulting in an increase of NBD-C_6_-SM (Fig. 3B). Moreover, while CERK knockdown caused a decrease in NBD-C1P, the knockdown of CERT—the protein that shuttles Cer from the ER to the Golgi—led to an increase in NBD-C_6_-C1P (Fig. 3C).

**Fig. 4:**
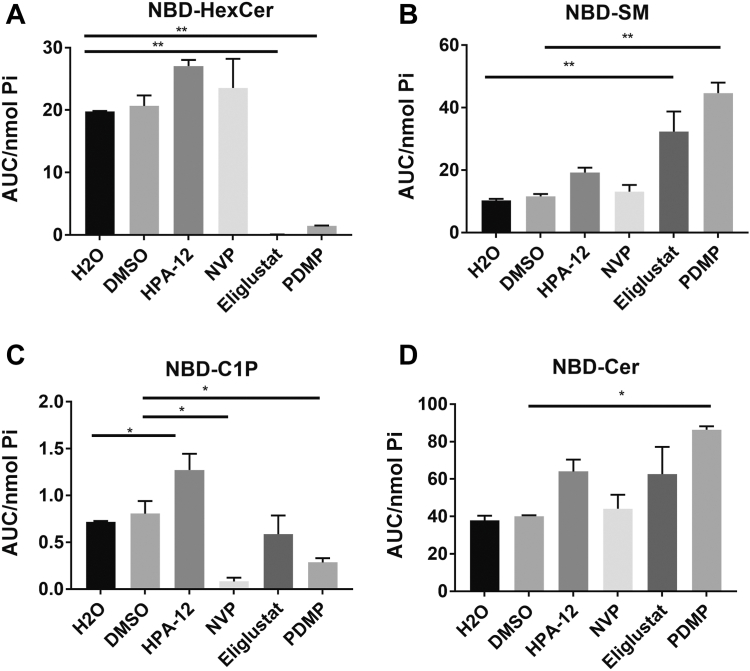
Measurement of NBD-sphingolipids in pharmacologically treated MCF7 cells. MCF7 cells were treated with 20 μM HPA-12, 500 nM NVP231, 50 nM eliglustat, 50 μM PDMP or the vehicle controls (H2O or DMSO) for 4 h. Cells were treated with 1 μM NBD-Cer for 1 h. Lipids were extracted and analyzed for (A) NBD-HexCer, (B) NBD-SM, (C) NBD-C1P, and (D) NBD-Cer by HPLC and fluorescence detection. Levels of NBD-sphingolipids were normalized to total lipid phosphate. Results are expressed as mean ± SEM of the AUC of detected peaks and are representative of at least three independent experiments. One-way ANOVA was used for statistics (∗ = *P* < 0.05, ∗∗ = *P* < 0.005). AUC, area under the curve; NBD, N-[6-[(7-Nitro-2-1,3-benzoxadiazol-4-yl)amino]dodecanoyl]; PDMP, 1-phenyl-2-decanoylamino-3-morpholino-1-propanol.

To consolidate these results, it was also important to show that assay conditions were sensitive enough to detect posttranslational inhibition of enzymes. Accordingly, MCF7 cells were subjected to pharmacological inhibition with NVP-231 (CERK), PDMP (GCS), eliglustat (GCS), or HPA-12 (CERT) prior to NBD-C_6_-Cer treatment (Fig. 4). Use of these inhibitors did not affect the localization of NBD- C_6_-Cer to the Golgi (supplemental Fig. S3). Inhibition with eliglustat and PDMP both nearly eliminated the production of NBD-C_6_-HexCer (Fig. 4A) as expected but also led to increased NBD-C_6_-SM (Fig. 4B), as was seen with GCS siRNA above. Notably, treatment with PDMP but not eliglustat also decreased the production of NBD-C_6_-C1P (Fig. 4C). This effect, in comparison to the downregulation of GCS, suggests that PDMP may have an inhibitory effect on CERK and is a promiscuous inhibitor (37, 38, 39, 40). NBD-C_6_-Cer increased with PDMP and a similar increase was observed with eliglustat although it was not significant (Fig. 4D). Treatment with NVP-231 inhibited the production of NBD-C_6_-C1P consistent with effects on CERK activity (Fig. 4C), whereas the CERT inhibitor HPA-12 led to an increase in NBD-C1P, as was seen with CERT siRNA (Fig. 4C), an expected result since CERT action is not required for Golgi localization of NBD-C_6_-Cer. Taken together, the data from siRNA knockdown and pharmacological inhibition of the key enzymes that metabolize NBD-C_6_-Cer at the Golgi show that coupling in situ NBD-C_6_-Cer tracing with our optimized HPLC analytical method can accurately measure changes of multiple NBD sphingolipids to recapitulate changes in the amount of total sphingolipid enzymes or enzymatic activities in the Golgi.

### Low-throughput screening of other inhibitors of the sphingolipid pathway on CERK

**Fig. 5:**
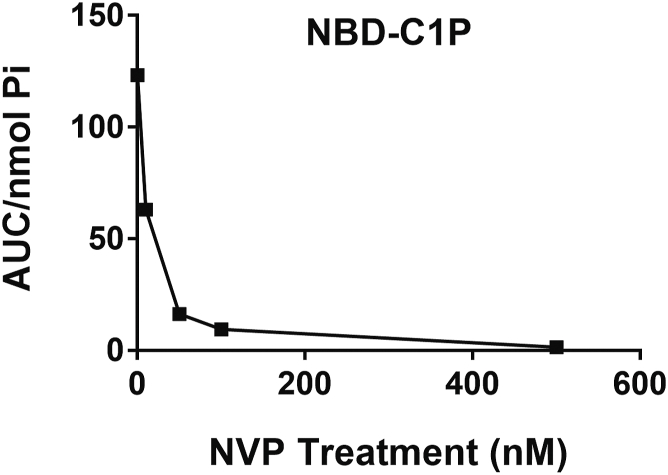
Measurements of NBD-C1P in MCF7 cells stably overexpressing CERK and inhibition with NVP-231. MCF7 cells stably overexpressing CERK were treated with varying doses of NVP-231 for 4 h. Cells were treated with 1 μM NBD-Cer for 1 h. Lipids were extracted and analyzed for NBD-C1P by HPLC and fluorescence detection. Basal levels of NBD-C1P were 10 times higher in the CERK overexpressing cells compared to their control (not shown). CERK, ceramide kinase; C1P, ceramide-1-phosphate; NBD, N-[6-[(7-Nitro-2-1,3-benzoxadiazol-4-yl)amino]dodecanoyl].

**Fig. 6:**
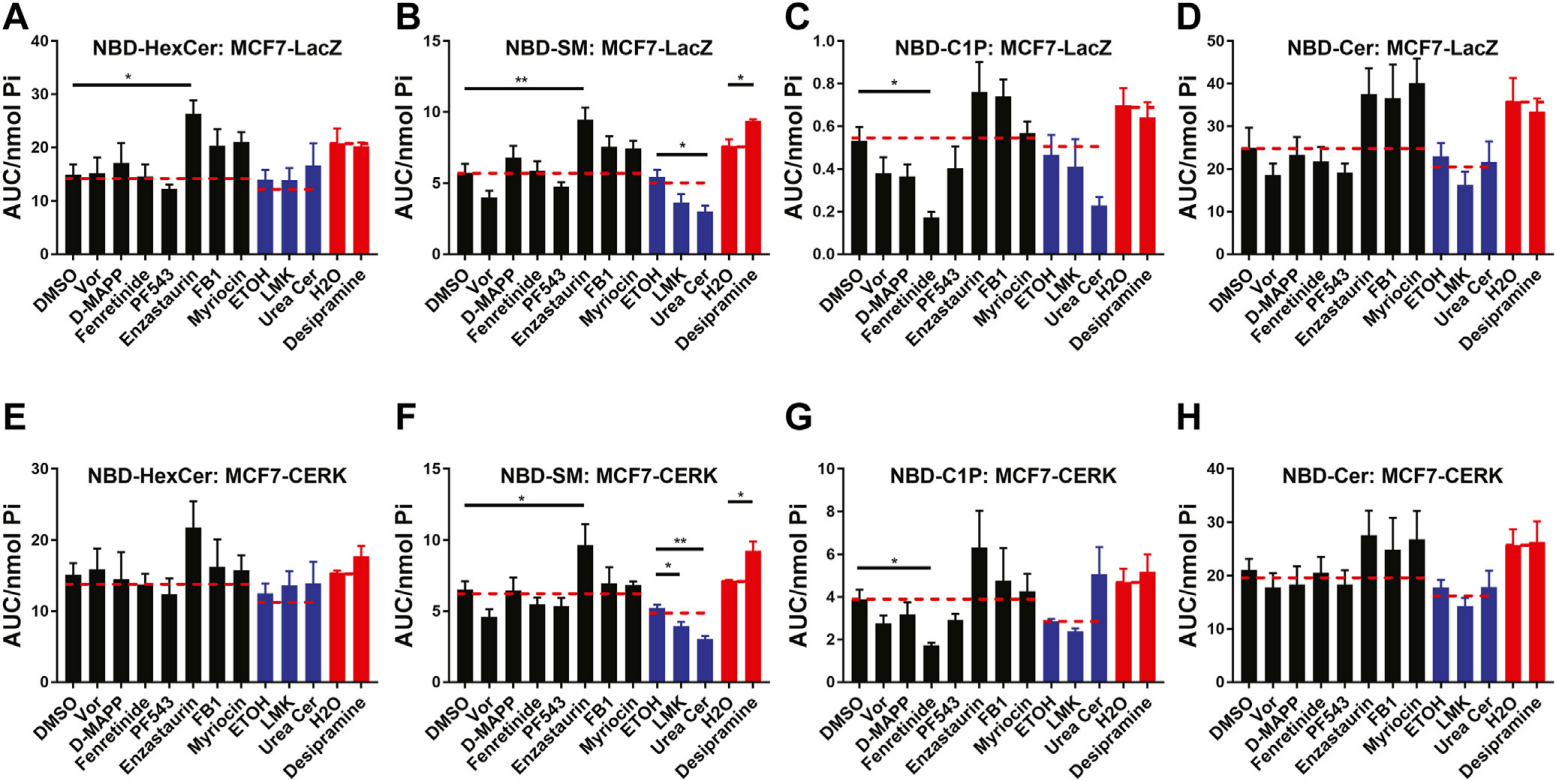
Evaluation of common inhibitors of sphingolipid enzymes using the NBD-Cer method. MCF7 LacZ or CERK overexpressing cells were treated with 1 μM enzastaurin, 2.5 μM LMK-235, 5 μM vorinostat, 5 μM urea ceramide, 10 μM D-MAPP, 10 μM desipramine, 100 nM myriocin, 50 μM fumonisin B1, 5 μM fenretinide, or 100 nM PF543 or their vehicle controls (DMSO, H2O, and EtOH) for 4 h. Cells were treated with 1 μM NBD-Cer for 1 h. Lipids were extracted in the LacZ control cells (A–D) or the CERK overexpressing cells (E–H) and analyzed for (A, E) NBD-HexCer, (B, F) NBD-SM, (C, G) NBD-C1P, and (D, H) NBD-Cer by HPLC and fluorescence detection. Levels of NBD-sphingolipids were normalized to total lipid phosphate. Results are expressed as mean ± SEM of the AUC of detected peaks and are representative of at least three independent experiments. One-way ANOVA was used for statistics (∗ = *P* < 0.05, ∗∗ = *P* < 0.005). *Black*, *blue*, and *red* colors represent inhibitors grouped with their respective vehicle controls. AUC, area under the curve; CERK, ceramide kinase; C1P, ceramide-1-phosphate; NBD, N-[6-[(7-Nitro-2-1,3-benzoxadiazol-4-yl)amino]dodecanoyl].

Given the unexpected effects of PDMP on CERK activity, we sought to deploy this assay to evaluate other commonly used inhibitors of sphingolipid metabolism. However, to advance this specific line of investigation, we had to optimize the detection of C1P. In all previous results, assays were performed in WT MCF7 cells, but while the measurements of NBD-C_6_-C1P were detectable, they nonetheless were very low. For a more accurate measurement of changes in C1P, MCF7 cells stably overexpressing CERK (MCF7-CERK) and counterpart controls (MCF7-LacZ) were generated using the lentiviral gateway system. Validation of MCF7-CERK cells showed generation of approximately ten times more NBD-C_6_-C1P than their LacZ counterparts (data not shown). Furthermore, a dose-response of NVP-231 (0–5 μM) established an IC50 of less than 10 nM in these MCF7-CERK cells (Fig. 5) confirming what was previously reported in the literature (41, 42). Indeed, the activity of CERK was almost entirely inhibited at 100 nM NVP-231. Subsequently, a variety of commonly used pharmacological inhibitors primarily involved with sphingolipid metabolism were evaluated including 4HPR (inhibitor of DES1), PF-543 (inhibitor of sphingosine kinase 1), urea ceramide (inhibitor of neutral ceramidase), myriocin (inhibitor of serine palmitoyltransferase), desipramine (indirect inhibitor of acid sphingomyelinase and acid ceramidase), fumonisin B1 (inhibitor of CerS), and D-MAPP (alkaline ceramidase). We also evaluated inhibitors not known to directly affect sphingolipid metabolism including enzastaurin (inhibitor of protein kinase C), vorinostat (histone deacetylases 1/2/3/6 inhibitor), and LMK-235 (histone deacetylases 4/5 inhibitor). Vorinostat and LMK-235 may indirectly affect sphingolipid metabolism through the epigenetic status of neutral sphingomyelinase-2 (43), which can also localize at the Golgi complex (44, 45, 46). A full list of inhibitors and the concentrations used are shown in Table 1. These inhibitors were used on MCF7-LacZ (Fig. 6A–D) to assess changes in the activity of the sphingolipid enzymes and in MCF7-CERK cells (Fig. 6E–H) to assess changes in cellular CERK activity more clearly. Most of these inhibitors (vorinostat, D-MAPP, PF-543, fumonisin B1, and myriocin) did not affect the NBD-sphingolipid levels in both the MCF7-LacZ and MCF7-CERK cells (Fig. 6). Enzastaurin increased NBD-C_6_-HexCer (Fig. 6A) with similar trends in MCF7-LacZ cells and MCF7-CERK cells (Fig. 6E), and similarly increased NBD-C_6_-SM in both cell types (Fig. 6B, F). C_6_-urea ceramide caused a decrease in NBD-C_6_-SM in both cell types (Fig. 6B, F) while LMK-235 decreased NBD-C_6_-SM primarily in MCF7-CERK cells (Fig. 6F) although a similar trend was observed in MCF7-LacZ cells (Fig. 6B). Finally, 4HPR inhibited NBD-C_6_-C1P production in both cell lines similar to the inhibition of NBD-C_6_-C1P seen above with PDMP (Fig. 6C, G). No significant changes were seen in NBD-C_6_-Cer in both cell lines (Fig. 6D, H).

**Table 1:** List of inhibitors

Inhibitor	Concentration	Enzyme Inhibited
PDMP	50 μM	GCS
Eliglustat	50 nM	GCS
HPA-12	20 μM	CERT
NVP-231	500 nM	CERK
Enzastaurin	1 μM	Protein kinase C
LMK-235	2.5 μM	Histone deacetylase 4/5
Vorinostat	5 μM	Histone deacetylases 1/2/3/6
Urea ceramide	5 μM	Neutral ceramidase
D-MAPP	10 μM	Acid ceramidase
Desipramine	10 μM	Acid sphingomyelinase
Myriocin	100 nM	Serine palmitoyltransferase
Fumonosin B1	50 μM	Ceramide synthases
Fenretinide	5 μM	Desaturase 1
PF543	100 nM	Sphingosine kinase 1

List of inhibitors screened on MCF7 cells and their intended target enzyme and the concentrations they were used to inhibit after 4 h.

### Evaluation of PDMP inhibition of CERK

**Fig. 7:**
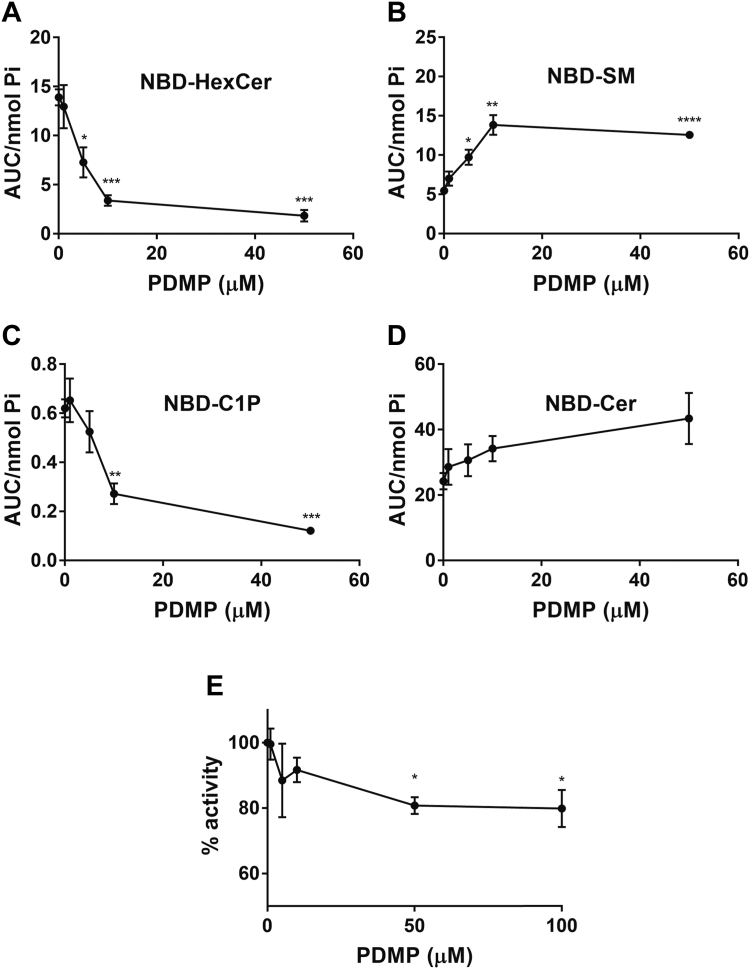
Inhibition of CERK by PDMP. MCF7 cells were treated with varying doses of PDMP for 4 h. Cells were treated with 1μM NBD-Cer for 1 h. Lipids were extracted and analyzed for (A) NBD-HexCer, (B) NBD-SM, (C)NBD-C1P, and (D) NBD-Cer by HPLC and fluorescence detection. Levels of NBD-sphingolipids were normalized to total lipid phosphate. Results are expressed as mean ± SEM of the AUC of detected peaks and are representative of at least three independent experiments. Varying doses of PDMP were incubated with purified CERK protein and analyzed for (E) CERK activity. Results are expressed as mean ± SEM of the AUC of C1P peak/total fluorescence relative to 0 μM PDMP and are representative of at least three independent experiments. Student’s *t* test was used for statistics (∗ = *P* < 0.05, ∗∗ = *P* < 0.005, ∗∗∗ = *P* < 0.0005, ∗∗∗∗ = *P* < 0.0001). AUC, area under the curve; CERK, ceramide kinase; C1P, ceramide-1-phosphate; NBD, N-[6-[(7-Nitro-2-1,3-benzoxadiazol-4-yl)amino]dodecanoyl]; PDMP, 1-phenyl-2-decanoylamino-3-morpholino-1-propanol.

To further evaluate inhibition of CERK by PDMP, dose-responses on MCF7-LacZ cells were conducted. For PDMP, a dose-response of 0–50 μM was used, and the cells were treated for 4 h prior to labeling as above (Fig. 7). As can be seen, there was no significant change in NBD-C_6_-Cer over the different concentrations of PDMP although values were trending upward at higher PDMP concentrations (Fig. 7D). In contrast, NBD-C_6_-HexCer decreased in a dose-dependent manner reaching almost maximal inhibition by 10 μM and diminished effects at higher concentrations (Fig. 7A). Importantly, PDMP inhibited formation of NBD-C_6_-C1P with a similar dose-response (Fig. 7C). It should also be noted that NBD-C_6_-SM increased as the concentration of PDMP increased (Fig. 7B), suggesting diversion of Golgi ceramide toward SM synthesis. Nonetheless, taken together, these results show that PDMP is nearly equipotent and effective against both GCS and CERK. To further assess the inhibition of CERK by PDMP, in vitro activity assays using purified CERK were performed at varying doses of PDMP. Surprisingly, the in vitro activity of CERK decreased only modestly with increasing concentration of PDMP (Fig. 7E) with a 20% decrease in activity seen at both 50 μM and 100 μM PDMP. This decrease is much less than the observed inhibition of NBD-C_6_-C1P production in cells. While this suggests PDMP can inhibit CERK activity directly to some degree, it also cannot fully explain the profound decrease in activity that was seen in cells.

### Evaluation of PDMP biological effects

**Fig. 8:**
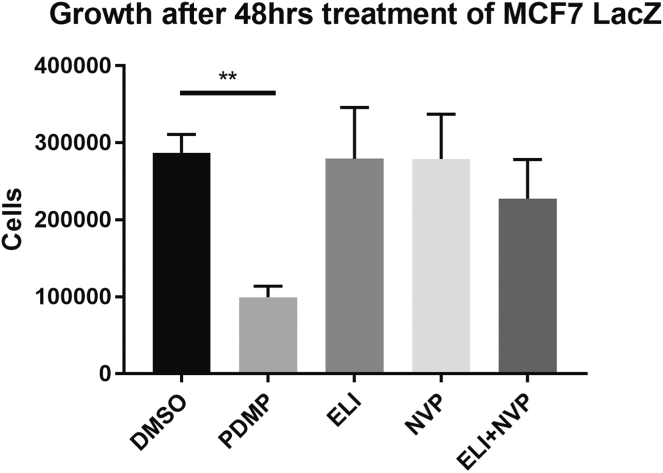
Effects of GCS inhibitors and NVP on cell growth. In total, 5 × 10^5^ cells MCF7 LacZ cells were plated and 24 h post plating were treated with 50 μM PDMP, 500 nM eliglustat, 500 nM NVP, or 500 nM eliglustat, and 500 nM NVP for 48 h. Total cell counts of LacZ control cells were measured by trypan blue staining. Results are expressed as mean ± SEM of the total cell count and are representative of at least three independent experiments. One-way ANOVA was used for statistics (∗∗ = *P* < 0.005). GCS, glucosylceramide synthase; PDMP, 1-phenyl-2-decanoylamino-3-morpholino-1-propanol.

PDMP is known to inhibit growth and cause cell death (47, 48, 49), although the role of inhibition of GCS in this process is not defined. Given the above results indicating that PDMP is a more promiscuous inhibitor of Golgi sphingolipid enzymes, it was important to evaluate if the prior reported biological effects of PDMP are a consequence of inhibition of GCS, CERK, or both. For this, cells were treated with the inhibitors eliglustat, NVP-231, or a combination of the two or with PDMP, and cell counts after 48 h of treatment were assessed (Fig. 8). Importantly, both of these are nM inhibitors of their targets and were used at concentrations that showed robust inhibition of their respective targets (Fig. 4A, C above). As expected, PDMP treatment led to a significant reduction in cell number after 48 h in MCF7 cells (Fig. 8). Treatments with eliglustat or NVP alone did not affect the number of MCF7 cells after 48 h, suggesting that inhibiting either GCS or CERK is not sufficient for reducing the cell count (Fig. 8). Interestingly, inhibiting both GCS and CERK also did not reduce the cell count (Fig. 8). These results suggest that the inhibition of GCS and CERK together is not sufficient to explain the growth-suppressing effects of PDMP.

### Evaluation of 4HPR inhibition of CERK

**Fig. 9:**
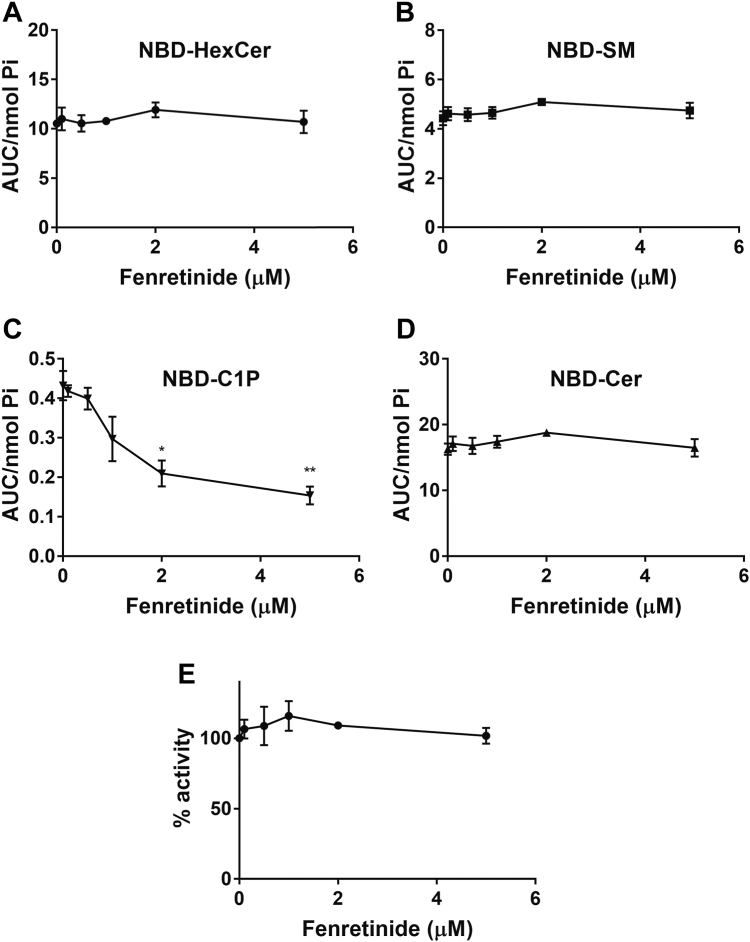
Dose-response of fenretinide reveals inhibition of CERK. MCF7 cells were treated with varying doses of fenretinide for 4 h. Cells were treated with 1 μM NBD-Cer for 1 h. Lipids were extracted and analyzed for (A) NBD-HexCer, (B) NBD-SM, (C)NBD-C1P, and (D) NBD-Cer by HPLC and fluorescence detection. Levels of NBD-sphingolipids were normalized to total lipid phosphate. Results are expressed as mean ± SEM of the AUC of detected peaks and are representative of at least three independent experiments. Varying doses of fenretinide were incubated with purified CERK protein and analyzed for (E) CERK activity. Results are expressed as mean ± SEM of the AUC of C1P peak/total fluorescence relative to 0 μM fenretinide and are representative of at least three independent experiments. Student’s *t* test was used for statistics (∗ = *P* < 0.05, ∗∗ = *P* < 0.005). AUC, area under the curve; CERK, ceramide kinase; C1P, ceramide-1-phosphate; NBD, N-[6-[(7-Nitro-2-1,3-benzoxadiazol-4-yl)amino]dodecanoyl].

The comparable effects of 4HPR and PDMP on NBD-C_6_-C1P levels seen above, as well as the broad use of 4HPR as a research tool and inhibitor of DES1, prompted us to investigate its effects on CERK activity further. For this, a similar dose-response of 4HPR (from 0 μM to 5 μM) was conducted, following the changes of NBD-sphingolipids after 4 h (Fig. 9). As can be seen, there were no significant changes observed in NBD-C_6_-Cer, NBD-C_6_-HexCer, or NBD-C_6_-SM. In contrast, singularly among the NBD-C_6_-Cer metabolites, NBD-C_6_-C1P trended downward as 4HPR concentration increased (Fig. 9C), reaching significant levels at 2 μM. Further investigations of the effects of 4HPR were performed by in vitro assay with purified CERK at varying doses of 4HPR. However, unlike with PDMP, 4HPR had no inhibitory effects on CERK in vitro (Fig. 9E). Taken together, these results confirm that 4HPR is able to inhibit in situ NBD-C_6_-C1P production but that this may be through indirect effects rather than through direct inhibition of CERK.

## Discussion

In this study, we have introduced a refined method for measuring and monitoring Golgi metabolism of ceramide. We established optimal dosage and acute time for NBD-C_6_-Cer treatment Cer uptake and metabolism into MCF7 cells. The respective NBD sphingolipid products of GCS, SMS1, and CERK—namely NBD-C_6_-HexCer, NBD-C_6_-SM, and NBD-C_6_-C1P—were identified using standards and were further validated with siRNA-mediated knockdown of the enzymes. We have further established the usefulness of this method to evaluate inhibitor efficacy for the target enzymes (CERK, SMSs, and GCS), and while screening various inhibitors of sphingolipid metabolism and other pharmacological agents, we discovered that PDMP and 4HPR were identified to have off-target inhibition of CERK in cells. This was previously unknown.

The first major conclusion of this study is that the current method is fit for monitoring activities of Golgi enzymes of ceramide metabolism. NBD-C_6_-Cer was incorporated and metabolized within 60 min to hexosylceramide (HexCer), sphingomyelin, and C1P. From the work of Pagano, NBD-C_6_-Cer is trafficked to the Golgi and appears in the Golgi within 30 min (50). Strong reductions in NBD-C_6_-HexCer, NBD-C_6_-SM, and NBD-C_6_-C1P following genetic knockdown or pharmacological inhibition of the responsible sphingolipid enzymes validated the identification and measurements of the complex sphingolipids. Taken together, these data show that Golgi metabolism of NBD-C_6_-HexCer, NBD-C_6_-SM, and NBD-C_6_-C1P are successfully monitored simultaneously in a cell-based assay for the first time. HPLC has been used to measure various NBD-sphingolipids individually. HPLC using NBD-C_6_-Cer has been used to measure the activity of GCS in vitro and in vivo (51, 52) and measure the activity of SMS (53, 54). GCS activity has also been measured in cell-based assays using NBD-C_6_-Cer with LC/MS/MS (9). Currently, CERK activity is measured through an in vitro assay that quantifies fluorescence of NBD-C_6_-C1P in a 96 well plate with TLC validation and has been suggested for use in situ (4). CERK activity is also determined by measurement of C1P by LC/MS/MS, but HPLC has not been used for measuring NBD-C_6_-C1P (55, 56). While our assay proves effective at simultaneously measuring CERK, GCS, and SMS activity, it should be noted that there are some limitations. For example, while HPLC is more accessible and cheaper than MS, the measurement of fluorescence is not as precise and quantitative as LC/MS/MS measurement of sphingolipids. This method may also be limited by the bulky nitrobenzyl moiety that provides the fluorescence. While Pagano showed that the NBD-C_6_-Cer is incorporated and metabolized similarly to the radiolabeled ceramide (3), there is a possibility that the bulkiness of the NBD moiety may change how ceramide is metabolized overall. However, the current data accurately reflect the changes in NBD-sphingolipids that are predicted to occur with the shown manipulation of the relevant sphingolipid enzymes, which would suggest that any changes to metabolism the NBD might cause is likely minimal. It is also important to note that this is a snapshot of NBD-C_6_-Cer metabolism in the Golgi in acute time points and not a reflection of overall steady-state levels.

The interconnected flux of sphingolipid metabolism can provide insight into how various genetic or pharmacological manipulations could affect sphingolipid metabolism. Our data measured NBD-sphingolipid levels after acute pharmacological enzymatic inhibition as well as following siRNA knockdown of select sphingolipid genes for 48 h. siRNA-mediated knockdown of CERK resulted in an increase of NBD-C_6_-SM formation, indicating a possible substrate competition with SMS1. This metabolic competition between CERK and SMS1 has been previously observed in Cos cells overexpressing CERK and in CERK KO BMDM cells using NBD-Cer (55). On the other hand, this was not observed with acute pharmacological inhibition of CERK. Also downregulation of SMS1 did not lead to an increase in NBD-C1P possibly because C1P is short lived as suggested by Bornancin’s group (55). As expected, downregulation or inhibition of CERT did not decrease synthesis of NBD-C_6_-SM or NBD-C_6_-C1P supporting that NBD-C_6_-Cer is not a substrate for CERT. It is unclear whether NBD-C_6_-Cer is delivered directly to the Golgi or brought elsewhere (ER) first. NBD-C_6_-Cer accumulates to the Golgi in a manner independent of protein trafficking from the ER in live cells and still accumulates to the Golgi in fixed cells (2). However, inhibition of Aur1 in yeast (the yeast ortholog of SMS) and inhibition of SMSs in mammalian cells results in a pattern similar to the ER, suggesting that Golgi staining of NBD requires SM synthesis (57, 58). It is known that CERT KO reduces endogenous SM synthesis which was not seen with NBD-C_6_-Cer, supporting that NBD-C_6_-Cer is not carried to the Golgi by CERT (55, 59). Studies of CERT would be better carried out utilizing Bodipy-Cer as it is a known substrate for CERT (55, 59). This does not rule out that NBD-C_6_-Cer is brought to the Golgi by vesicular trafficking. Nonetheless, NBD-C_6_-Cer is delivered to the Golgi and metabolized by the sphingolipid enzymes. As already reported, decrease of GCS (inhibition or downregulation) resulted in increased production of NBD-C_6_-SM (60). These metabolic changes support the validity of our method.

The second major conclusion of this study is that this method can reveal off-target effects of inhibitors as screens of widely used sphingolipid inhibitors revealed that PDMP and 4HPR are both able to decrease in situ CERK activity. While PDMP has been widely used and cited as a GCS inhibitor, it is not very specific, having been found to inhibit ceramide glycanase and lysosomal phospholipase A2, which can acylate ceramide (39, 40) and have effects on calcium homeostasis (38) and thymidine transport (37). Similarly, 4HPR is a direct inhibitor of DES1, but other changes in sphingolipid metabolism have also been identified. For example, 4-HPR has been reported to indirectly enhance de novo synthesis of ceramide through increased activity of serine palmitoyl transferase and ceramide synthases (61, 62, 63). While both PDMP and 4HPR showed decrease of CERK activity in situ, in vitro assays revealed that PDMP inhibited CERK although at a much smaller degree and that 4HPR did not inhibit CERK. The mechanisms by which CERK activity is being modulated by PDMP and 4HPR in situ have yet to be fully identified.

The many targets of PDMP have implications for its biological effects and suggest significant caution in use of PDMP as an inhibitor of GCS, particularly given that distinct phenotypes arise with PDMP treatment when compared to other more specific GCS inhibitors such as eliglustat. For example, PDMP has been well-documented to resensitize drug-resistant cancers to chemotherapeutic treatment, mostly thought to be due to ceramide accumulation but also through inhibition of P-gp (64, 65, 66, 67, 68). However, multidrug resistance due to P-gp encoded by the *MDR1* gene was modulated only by the PDMP family of inhibitors but not by the iminosugar GCS inhibitors, which includes eliglustat (66, 69, 70, 71). Another phenotype recently reported was that PDMP induced lysosomal lipid accumulation and mammalian target of rapamycin inactivation, but these effects were also not seen with more specific GCS inhibitors (47, 72). Outside of questionable specificity, a further relevant variable to consider is that many studies use PDMP treatment with doses at 50 μM and above, whereas our studies here show maximal inhibitory effects of PDMP at 10 μM and lower (at least in MCF7 cells). This disconnect between inhibitor efficacy against their target with what are “expected’ biological responses to target inhibition can often lead to excessive inhibitor doses being utilized. In our dose-response of PDMP, we newly observed PDMP to also inhibit NBD-C_6_-C1P formation by CERK in a dose-dependent manner and well within the range of concentrations that have been used previously. Cell cycle arrest is a biology well-associated with PDMP (47, 49); however, the growth suppressive effects of PDMP seen in our results were not replicated by eliglustat which is a more specific inhibitor for GCS. Cell cycle arrest is also caused by the CERK inhibitor NVP-231 (41). However, we did not see any changes in cell number caused by NVP-231, albeit the concentrations used in our study were much lower than those used by Pastukov *et al.*—but were nonetheless effective at inhibiting CERK activity in our system. Furthermore, while PDMP inhibits CERK, the combined inhibition of CERK and GCS was not sufficient to replicate PDMP’s biological effects since the combination of eliglustat and NVP-231 did not affect cell number. PDMP’s effects are likely driven through additional targets, yet to be defined. Nonetheless, the biological effect of PDMP is inconsistent with eliglustat treatment and not due to inhibition of GCS.

In summary, we have advanced and tightened an assay using NBD-C_6_-Cer and HPLC to monitor Golgi sphingolipid metabolism in intact cells. In addition, PDMP and 4HPR were shown to inhibit CERK activity in situ although the mechanisms are not fully known, advancing knowledge of off-target effects of known inhibitors.

## Data availability

The data supporting the conclusions of this article are included within the article and supplementary materials.

## Supplemental data

This article contains supplemental data.

## Conflict of interest

The authors declare that they have no conflicts of interest with the contents of this article.

## Supplemental data

Supplemental Figure 1 HPLC Chromatogram and NBD-Standards. MCF7 cells were treated with (A) DMSO or (B) NVP and subject to 1μM NBD-Cer treatment for 1 h. Lipids were extracted by HPLC and fluorescence detection and the representative chromatograms were shown. The insets show a zoomed-in view of NBD-C1P in these cells. NBD-standards for (C) ceramide, (D) glucosylceramide, (E) sphingomyelin, and (F) C1P were run and their chromatograms are shown.

Supplemental Figure 2 RT-PCR validation of siRNA. MCF7 cells were transfected for 48 h with all star (AS) scrambled siRNA or siRNA for CERK, CERT, GCS, SMS1, SMS2, or SMS1+2. mRNA was extracted and converted to cDNA and analyzed for (A) CERT (B) CERK (C) GCS (D) SMS1 (E) SMS2 by qRT-PCR using taqman probes. Results are expressed as mean ± SEM of the mean normalized expression and are representative of at least three independent experiments. One way ANOVA was used for statistics (∗ = *P* < 0.05, ∗∗ = *P* < 0.005, ∗∗∗ = *P* < 0.0005, ∗∗∗∗ = *P* < 0.0001).

Supplemental Figure 3 Live Cell imaging of NBD-Cer with Inhibitors. MCF7 cells were plated and treated with (A) DMSO, (B) NVP-231, (C) PDMP, (D) Eliglustat for 4 h. Cells were treated with 1μM NBD-Cer (Green) for 1 h and stained with Hoechst (Blue) to visualize the nuclei for live cell imaging. Cells were imaged with a confocal microscope at x63 magnification.
