# Supplementary figures and images for: A comprehensive measure of Golgi sphingolipid flux using NBD C6-ceramide: evaluation of sphingolipid inhibitors
Source: J Lipid Res. 2024 Jun 24;65(8):100584. doi: 10.1016/j.jlr.2024.100584 (PMC11326893; doi:10.1016/j.jlr.2024.100584)

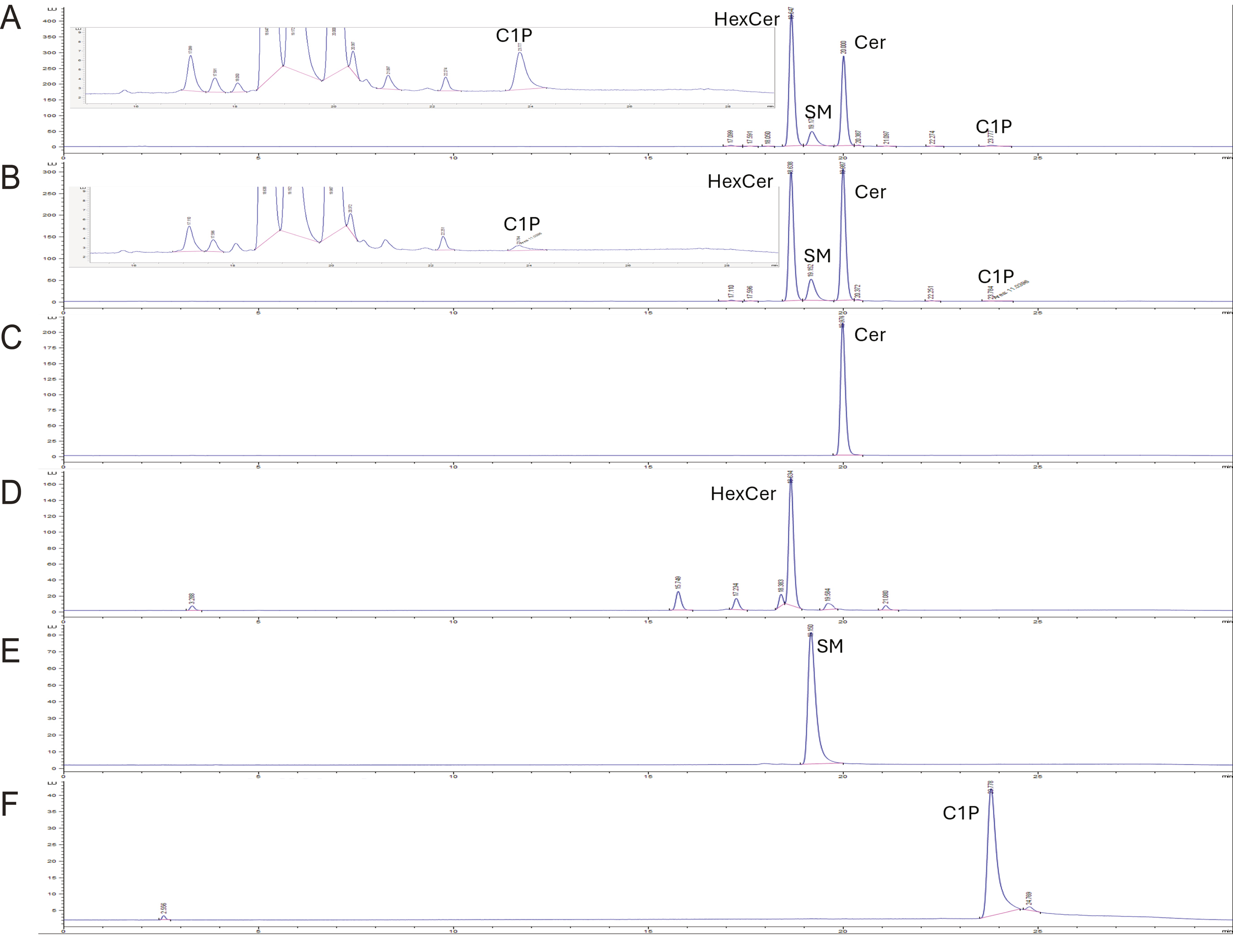

Supplement: Supplemental Figure 1 — HPLC Chromatogram and NBD-Standards. MCF7 cells were treated with (A) DMSO or (B) NVP and subject to 1μM NBD-Cer treatment for 1 h. Lipids were extracted by HPLC and fluorescence detection and the representative chromatograms were shown. The insets show a zoomed-in view of NBD-C1P in these cells. NBD-standards for (C) ceramide, (D) glucosylceramide, (E) sphingomyelin, and (F) C1P were run and their chromatograms are shown. [file figs1.jpg]

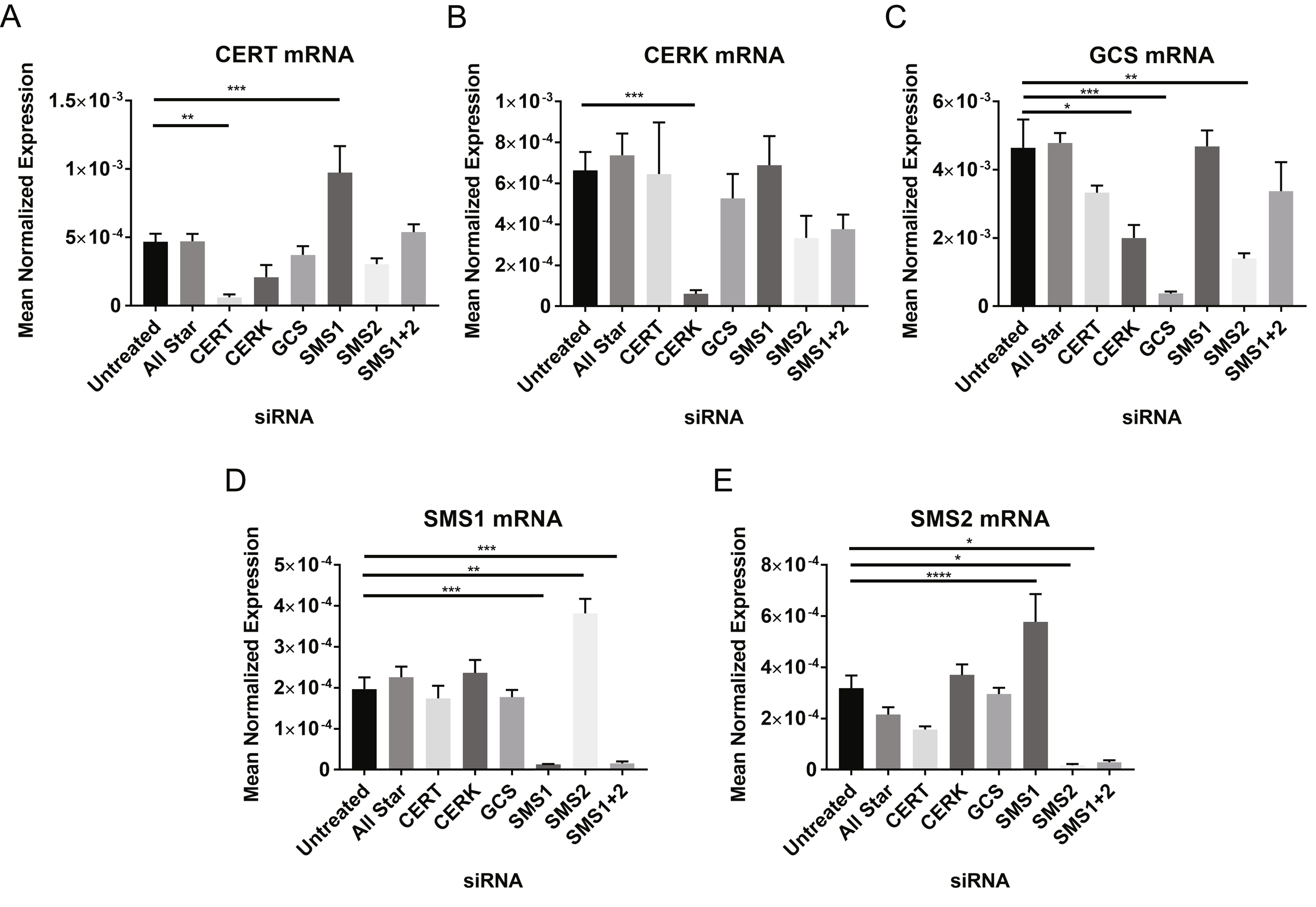

Supplement: Supplemental Figure 2 — RT-PCR validation of siRNA. MCF7 cells were transfected for 48 h with all star (AS) scrambled siRNA or siRNA for CERK, CERT, GCS, SMS1, SMS2, or SMS1+2. mRNA was extracted and converted to cDNA and analyzed for (A) CERT (B) CERK (C) GCS (D) SMS1 (E) SMS2 by qRT-PCR using taqman probes. Results are expressed as mean ± SEM of the mean normalized expression and are representative of at least three independent experiments. One way ANOVA was used for statistics (∗ = P < 0.05, ∗∗ = P < 0.005, ∗∗∗ = P < 0.0005, ∗∗∗∗ = P < 0.0001). [file figs2.jpg]

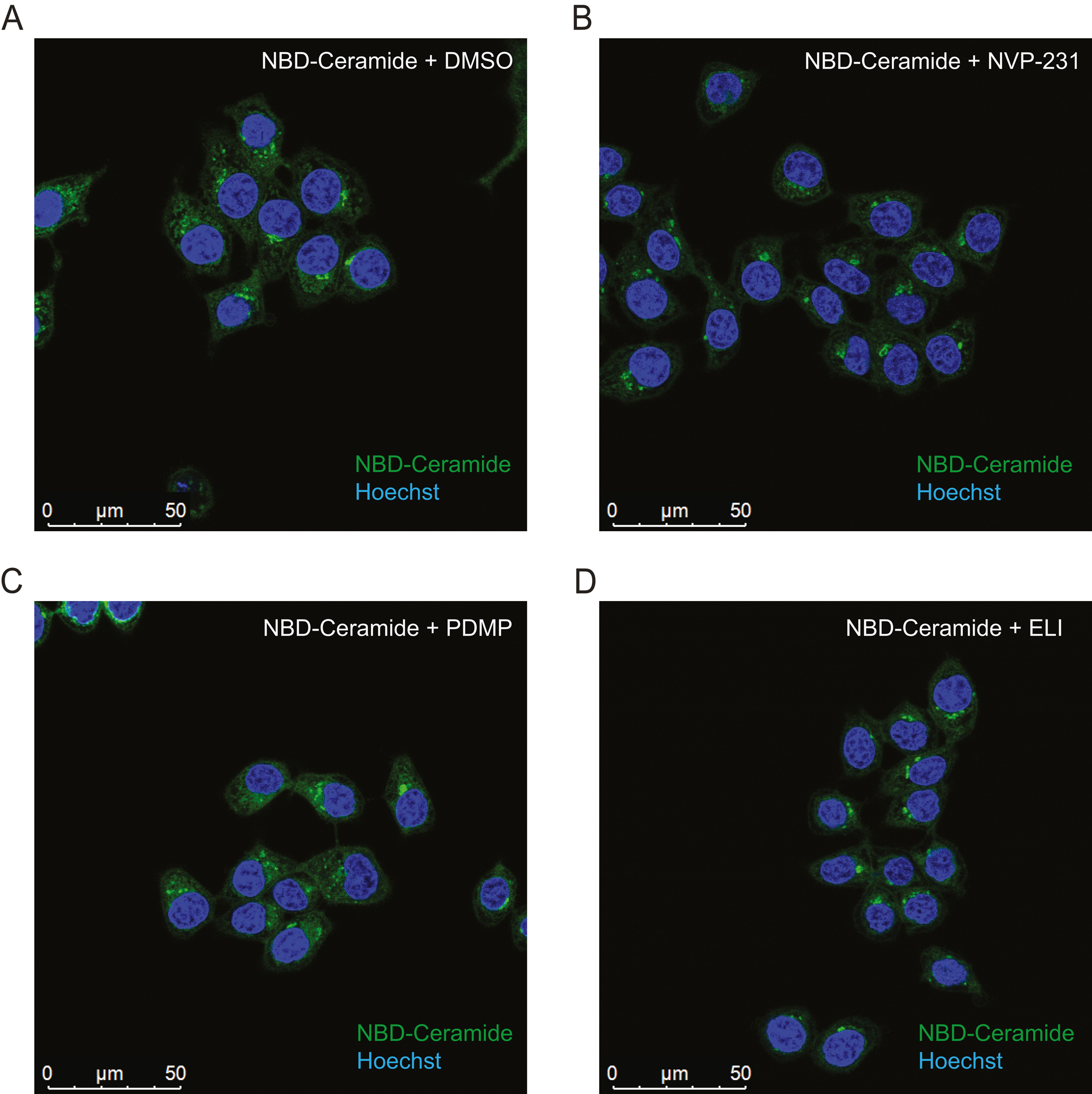

Supplement: Supplemental Figure 3 — Live Cell imaging of NBD-Cer with Inhibitors. MCF7 cells were plated and treated with (A) DMSO, (B) NVP-231, (C) PDMP, (D) Eliglustat for 4 h. Cells were treated with 1μM NBD-Cer (Green) for 1 h and stained with Hoechst (Blue) to visualize the nuclei for live cell imaging. Cells were imaged with a confocal microscope at x63 magnification. [file figs3.jpg]
